# Ethnomedicines used in Trinidad and Tobago for reproductive problems

**DOI:** 10.1186/1746-4269-3-13

**Published:** 2007-03-15

**Authors:** Cheryl Lans

**Affiliations:** 1 BCICS, University of Victoria, British Columbia, V8W 2Y2, Canada

## Abstract

**Background:**

Throughout history women have tried to control or enhance their fertility using herbal remedies, with various levels of societal support. Caribbean folk medicine has been influenced by European folk medicine, either through the early Spanish and French settlers or through the continuous immigration of Spanish-speaking peoples from Venezuela. Some folk uses are ancient and were documented by Galen and Pliny the Elder.

**Methods:**

Thirty respondents, ten of whom were male were interviewed from September 1996 to September 2000. The respondents were obtained by snowball sampling, and were found in thirteen different sites, 12 in Trinidad (Paramin, Talparo, Sangre Grande, Mayaro, Carapichaima, Kernahan, Newlands, Todd's Road, Arima, Guayaguayare, Santa Cruz, Port of Spain and Siparia) and one in Tobago (Mason Hall). Snowball sampling was used because there was no other means of identifying respondents and to cover the entire islands. The validation of the remedies was conducted with a non-experimental method.

**Results:**

Plants are used for specific problems of both genders. *Clusea rosea*, *Urena sinuata *and *Catharanthus roseus *are used for unspecified male problems. *Richeria grandis *and *Parinari campestris *are used for erectile dysfunction.

*Ageratum conyzoides*, *Scoparia dulcis*, *Cucurbita pepo*, *Cucurbita maxima*, *Gomphrena globosa *and *Justicia pectoralis *are used for prostate problems.

The following plants are used for childbirth and infertility: *Mimosa pudica*, *Ruta graveolens*,

*Abelmoschus moschatus*, *Chamaesyce hirta*, *Cola nitida*, *Ambrosia cumanenesis*, *Pilea microphylla*, *Eryngium foetidum*, *Aristolochia rugosa*, *Aristolochia trilobata*, *Coleus aromaticus*, *Laportea aestuans *and *Vetiveria zizanioides*.

The following plants are used for menstrual pain and unspecified female complaints:

*Achyranthes indica*, *Artemisia absinthium*, *Brownea latifolia*, *Eleutherine bulbosa*, *Hibiscus rosa-sinensis*, *Eupatorium macrophyllum*, *Justicia secunda*, *Parthenium hysterophorus*, *Wedelia trilobata*, *Abelmoschus moschatus*, *Capraria biflora*, *Cordia curassavica*, *Croton gossypifolius*, *Entada polystachya*, *Leonotis nepetaefolia*, *Eryngium foetidum*, *Aristolochia rugosa, Aristolochia trilobata *and *Ambrosia cumanenesis*.

**Conclusion:**

Native Caribbean plants have been less studied that those from Africa, India and Europe. *Chamaesyce hirta *has scientific support but as a diuretic. Other plants with level 3 validity for reproductive issues are: *Achyranthes indica, Coleus aromaticus, Hibiscus rosa-sinesis, Parthenium hysterophorus *and *Ruta graveolens*. The non-experimental validation method can be used to advise the public on which plants are safe, effective and useful, and which are not; pending clinical trials. This is especially important since so few clinical trials are conducted on Caribbean plants.

## Background

Throughout history women have tried to control or enhance their fertility with various levels of societal support. Many herbal remedies are traditionally used as contraceptives (to prevent ovulation or fertilisation), abortifacients (to prevent implantation), emmenagogues (to stimulate uterine flow) or oxytocics (to stimulate uterine contractions, particularly to promote labour) [1]. By 1988, the Natural Products Alert database had recorded 4,410 plants used as emmenagogues, 2,630 as abortives and 1,249 as contraceptives [1]. Women are increasingly turning to fertility-enhancing plants to combat against the negative effects of industrial pollutants on fertility [2]. This increases the need and even the ethical imperative for further scientific study on medicinal plants.

There are indications that even in developed countries such as Australia, women do not feel well served by what is currently available. In one Australian study seventy-two women, aged 18 to 50 years were interviewed and they claimed to be dissatisfed with contraceptive choices including their side effects [3]. Younger women were more accepting of medical opinion, while many older women rejected medical interference in contraceptive decisions [3]. There have also been reports of women in some developing countries unknowingly receiving a tetanus vaccine laced with the anti-fertility drug human chorionic gonadotropin (hCG) [4]; indicating that their individual interests were overridden by scientific authorities and that they need to be more proactive in maintaining their own health.

The Mount Hope Women's Hospital opened in Trinidad in 1981. It provides general access to standardised obstetric care for the general public. This is a tertiary care University of the West Indies-affiliated institution which receives referral of high and low risk pregnancies from other clinics, hospitals and doctors. A 1998 paper reported that for the Hospital's first 16 years of operation there were 33 obstetric deaths and 89, 286 mothers of live births or stillbirths, giving a maternal mortality rate of 36.9 per 100 000 births [5]. Thirty-two deaths were directly related to childbearing. Pregnancy-induced hypertension accounted for 17 deaths (eclampsia and severe pre-eclampsia). Antenatal care was defined as adequate or substandard. Avoidable factors were identified under three categories: poor patient compliance, faulty clinical management and administrative failure in the provision of medical facilities [5].

A more recent 6-year prospective perinatal audit at the Mount Hope Women's Hospital in Trinidad was conducted in order to determine foetal outcome, and the common causes of foetal and early neonatal deaths [6]. Of a total of 30,987 births, there were 469 stillbirths and 391 early neonatal deaths, producing a perinatal mortality rate of 27.7 per 1000 total births. Stillbirths resulted from the hypertensive disorders of pregnancy, abruptio placentae, diabetes mellitus, intrapartum foetal distress and lethal congenital anomalies [6]. Neonatal deaths were caused by respiratory distress syndrome (57.8%), birth asphyxia (22.2%) and sepsis (13.5%) [6]. Another study of early onset Group B streptococcal (GBS) infection in neonates at the Mount Hope Women's Hospital over the period 1996–97 found that the incidence of early onset neonatal GBS sepsis was five to six times higher than that reported in the USA and UK [7].

A World Health Organization (WHO) study showed that in Latin America and the Caribbean, hypertensive disorders were responsible for the most maternal deaths (point estimate 25.7%, range 7.9–52.4; ten datasets, 11 777 deaths). Abortion deaths were the highest in Latin America and the Caribbean (12%), and were as high as 30% of all deaths in some countries in the region. Deaths due to sepsis were higher in Latin America and the Caribbean (odds ratio 2.06) than in developed countries [8].

These studies show that a reliance on midwife-supported home births and women's traditional knowledge for unremarkable pregnancies should be considered so that women have more control over their own fertility which may result in better patient compliance and would reduce the burdens on the public hospitals allowing more time and resources to be spent on problematic pregnancies. It may also improve the standards of antenatal care that pregnant women receive and reduce the number of abortion-related deaths. This approach has already been proven in Jamaica [9]. The Victoria Jubilee (Lying-In) Hospital (VJH) in Jamaica promoted antenatal screening and selective booking for hospital birth of mothers considered unsuitable for home delivery. These included primigravidae, grandmultiparae, abnormal presentations, multiple pregnancies and women with previous obstetric problems. 'Normal' women were referred to community midwives [9]. The community midwife's role was expanded in the 1970s to include provision of family planning, antenatal, post-natal and child welfare services as well as attending home births. This integration into the community health team provided an avenue for midwives' continuing education, direct supervision and job security [9].

Previous research has shown that Caribbean women and Creoles have always used bitter herbs to control their fertility [10-13]; some of this knowledge may have been passed on to the current generation. Plants used before the 1950s were lignum vitae (*Guaiacum officinale*), seed under leaf (*Phyllanthus niruri*), gully root (*Petiveria alliacea*) and more poisonous purges like oleander (*Nerium oleander*) and mudar (*Calotropis procera*) [11]. Native Americans may have been the first to use lignum vitae as antiseptics, for syphilis and as stimulants [11].

Caribbean traditional reproductive health care focuses equally on the pregnancy, parturition and the postpartum period [13]. Research in Jamaica illustrated that birth, defecation and menstruation are defined traditionally as cleansing processes [13]. After births or miscarriages, mild purgatives are given to induce the quick delivery of the placenta and pregnancy-related waste matter through the vagina [13]. Emmenagogues are used to restore the menses, to "clean out" the womb, and to restore vitality after pregnancy [13]. All purgatives are classified as a "washout" and many women use "washout" ingredients as emmenagogues [10-13].

Latin American and Caribbean women also choose plants for reproductive conditions based on the properties that correspond to the hot-cold valence, irritating action, emmenagogic, oxytocic, anti-implantation and/or abortifacient effects [12]. Activities, food and medicines are classified in various ethnomedicinal systems as hot or cold. The hot-cold valence in this context refers to the traditional belief that heat opens the body and facilitates the blood's free flow, whereas cold causes the blood to stop flowing and clog the arteries, veins and womb [14]. One cause of infertility is described as "cold in the uterus" and fertility enhancers are considered to be "hot" [12,15]. In Mexico infertility in women is considered a "cold" illness and "hot" remedies are prescribed [16]. Uteroactive plants used in Mexico are described in metaphorical terms of "warming" or "irritating" [17]. "Warming" the body, blood and womb, causes the womb to "open" to release detained menstrual flow or expel a full – term foetus or unwanted conceptus [17]. "Irritating" plants "open" the uterus and stimulate contractions that will release blocked menstrual blood or push out a full – term foetus or unwanted conceptus [17].

This paper presents the plants used for reproductive purposes in Trinidad and Tobago. Plants were used for unspecified male problems, for erectile dysfunction and prostate problems. The plants used to address women's reproductive problems were used mainly for infertility, menstrual pain and childbirth. Unlike a previous publication on plants used for reproductive problems in Trinidad and Tobago [18], these plants had no comparable use for animals.

## Methods

This study adhered to the research guidelines and ethical protocols of Wageningen University in the Netherlands. Thirty respondents, ten of whom were male were interviewed from September 1996 to September 2000 [19]. The respondents were obtained by snowball sampling, and were found in thirteen different sites, 12 in Trinidad (Paramin, Talparo, Sangre Grande, Mayaro, Carapichaima, Kernahan, Newlands, Todd's Road, Arima, Guayaguayare, Santa Cruz, Port of Spain and Siparia) and one in Tobago (Mason Hall). Snowball sampling was used because there was no other means of identifying respondents. The chief objective of the sampling method was to identify knowledgeable respondents; no priority was given to extrapolating the data to the wider population to establish prevalence of use. No statistical analysis is applied to the data since this would have required the use of a random sample thus increasing the risk of not identifying knowledgeable respondents.

Twenty respondents were interviewed once, the other ten (who were healers) were interviewed three or four times. Healers were also asked to reconstruct the circumstances and contexts of the plant uses so that the means of administration of the plants could be identified. No interview schedule of questions was used but a more qualitative, conversational technique. Plants were collected when available to verify that the common names used by each respondent were the same in each ethnic group as those recorded in the literature. The majority of the plants were identified at the Herbarium of the University of the West Indies but voucher samples were not deposited. This ethnomedicinal study was part of a larger research project on ethnoveterinary medicine [11,18].

### Validation of practices

A preliminary validation of ethnomedicinal practices ensures that clinical trials are not wasted on plants that are used solely for cultural or religious reasons. The validation of the remedies was conducted with a non-experimental method [11,18,19]. This method consists of:

1. obtaining an accurate botanical identification,

2. determining whether the folk data can be understood in terms of bioscientific concepts and methods,

3. searching the chemical/pharmaceutical/pharmacological literature for the plant's known chemical constituents and to determine the known physiological effects of either the crude plant, related species, or isolated chemical compounds that the plant is known to contain. This information is used to assess whether the plant use is based on empirically verifiable principles or whether symbolic aspects of healing are of greater relevance. If ethnobotanical data, phytochemical and pharmacological information supports the folk use of a plant species it can be grouped into the validation level with the highest degree of confidence.

Four levels of validity were established [19]:

1. If no information supports the use it indicates that the plant may be inactive; or no research has been done on the plant.

2. A plant (or closely related species of the same genus), which is used in geographically or temporally distinct areas in the treatment of similar illnesses, attains the lowest level of validity, if no further phytochemical or pharmacological information validates the popular use. Use in other areas increases the likelihood that the plant is active against the illness.

3. If in addition to the ethnobotanical data, phytochemical or pharmacological information also validates the use in Trinidad, the plant may exert a physiological action on the patient and is more likely to be effective than those at the lowest level of validity.

4. If ethnobotanical [20], phytochemical and pharmacological data supports the folk use of the plant, it is grouped in the highest level of validity and is most likely an effective remedy.

A comparable validation process was used to examine the plants used by traditional healers of ancient Persia to induce abortions [21]. The authors evaluated the validity and the efficacy of the plants used by (1) comparing other reported uses of these plants in traditional medicine, (2) investigating the medical and pharmacological literature on the medicinal properties of the plant species used, and (3) investigating the reported cytotoxic effects of compounds prevalent in these plants.

## Results

*Mimosa pudica *was used by one midwife to unwrap the cord from around an unborn baby's neck. Two plant tops were tied crossways, put in a pot and drawn. It was claimed that fifteen minutes after the pregnant woman drank the tisane the baby gave a flip. However a caesarean was still needed because the baby's due date had past.

### Plants used for reproductive problems

Forty-two plants are used for reproductive problems of men and women. The term "man's waist pain" was not explained. The plants used for "man's disease" and "man's waist pain" were *Catharanthus roseus*, *Urena sinuata and Clusea rosea. Parinari campestris *and *Richeria grandis *are used for erectile dysfunction. Prostate problems are treated with *Ageratum conyzoides*, *Scoparia dulcis*, *Cucurbita pepo*, *Cucurbita maxima*, *Gomphrena globosa *and *Justicia pectoralis*.

Plants used for abortions are *Aristolochia rugosa*, *Aristolochia trilobata*, *Ambrosia cumanenesis *and *Cocos nucifera*.

Unspecified female complaints ae treated with *Achyranthes indica*, *Artemisia absinthium*, *Brownea latifolia*, *Eleutherine bulbosa*, *Hibiscus rosa-sinensis*, *Eupatorium macrophyllum*, *Justicia secunda*, *Parthenium hysterophorus*, *Wedelia trilobata*, *Abelmoschus moschatus *and *Ageratum conyzoides*. *Desmodium canum *is used for venereal diseases and *Nopalea cochinellifera *is used for menopause and hot flashes. The following plants are used for infertility and inflammations: *Chamaesyce hirta*, *Cola nitida*, *Ruta graveolens*, *Commelina elegans*, *Ambrosia cumanenesis *and *Pilea microphylla*.

The plants used for menstrual pain are: *Aristolochia rugosa*, *Aristolochia trilobata*

*Ruta graveolens*, *Ambrosia cumanenesis*, *Capraria biflora*, *Cordia curassavica*, *Croton gossypifolius*, *Entada polystachya*, *Leonotis nepetaefolia *and *Eryngium foetidum*. Plants used for childbirth, to shorten labour and remove the placenta are *Mimosa pudica*,

*Ruta graveolens*, *Abelmoschus moschatus*, *Aristolochia rugosa*, *Aristolochia trilobata*, *Coleus aromaticus*, *Laportea aestuans*, *Vetiveria zizanioides*, *Abelmoschus moschatus *and *Eryngium foetidum*. The ethnomedicinal plants used in Trinidad and Tobago for reproductive problems are summarised in Table 1. The validation of the plants is summarised in Table 2.

**Table 1:** Ethnomedicinal plants used for reproductive problems in Trinidad and Tobago

Scientific name	Family	Common name	Part used	Use
*Abelmoschus moschatus*	Malvaceae	Gumbo musque	Leaves, seeds	Female complaints, remove placenta
*Achyranthes indica*	Amaranthaceae	Man better man		Female complaints
*Ageratum conyzoides*	Asteraceae	Z'herbe á femme		Prostate, Female complaints
*Ambrosia cumanenesis*	Asteraceae	Altamis	3-inch	Inflammation, abortion, Menstrual pain
*Aristolochia rugosa*, *Aristolochia trilobata*	Aristolochiaceae	Mat root, anico	Root	Remove placenta, abortion, menstrual pain
*Artemisia absinthium*	Asteraceae	Wormwood		Female complaints
*Brownea latifolia*	Fabaceae	Cooper hoop	Flower, leaves	Female complaints
*Capraria biflora*	Scrophulariaceae	Du thé pays	Leaves	Menstrual pain
*Catharanthus roseus*	Apocynaceae	White Periwinkle		"Man's disease"
*Chamaesyce hirta*	Euphorbiaceae	Malomay		Infertility
*Clusea rosea*	Clusiaceae	Matapal	Bark	Bark belt for "man's waist pain"
*Cocos nucifera*	Arecaceae	Coconut	Shell	Abortion
*Cola nitida*	Sterculiaceae	Obie seed	Seed	Infertility
*Coleus aromaticus*	Lamiaceae	Spanish thyme	Leaves	Shorten labour
*Commelina elegans*	Commelinaceae	Water grass	Plant	Douche
*Cordia curassavica*	Boraginaceae	Black sage	Leaves	Menstrual pain
*Croton gossypifolius*	Euphorbiaceae	Bois sang	Leaves	Menstrual pain
*Cucurbita pepo*, *Cucurbita maxima*	Cucurbitaceae	Pumpkin		Prostate problems
*Desmodium canum*	Fabaceae	Sweet heart bush	Root	Venereal diseases
*Eleutherine bulbosa*	Iridaceae	Dragon blood	Bulb	Female complaints
*Entada polystachya*	Fabaceae	Mayoc chapelle	Twigs	Menstrual pain
*Eryngium foetidum*	Apiaceae	Chadron bénée	Leaves	Menstrual pain, remove placenta, shorten labour
*Eupatorium macrophyllum*	Asteraceae	Z'herbe chatte		Female complaints
*Gomphrena globosa*	Amaranthaceae	Bachelor button		Prostate problems
*Hibiscus rosa-sinensis*	Malvaceae	Hibiscus	Flowers	Female complaints
*Justicia pectoralis*	Acanthaceae	Carpenter grass	Leaves	Prostate problems
*Justicia secunda*	Acanthaceae	St. John's bush		Female complaints
*Laportea aestuans*	Urticaceae	Red stinging nettle	Leaves	Shorten labour
*Leonotis nepetaefolia*	Lamiaceae	Shandileer		Menstrual pain
*Mimosa pudica*	Fabaceae	Mese marie		Childbirth
*Nopalea cochinellifera*	Cactaceae	Rachette	Joint	Menopause, hot flashes
*Parinari campestris*	Chrysobalanaceae	Bois bandé	Bark	Erectile dysfunction
*Parthenium hysterophorus*	Asteraceae	White head broom		Female complaints
*Pilea microphylla*	Urticaceae	Du thé bethelmay	Leaves	Inflammation, womb cleanser
*Richeria grandis*	Euphorbiaceae	Bois bandé	Bark	Erectile dysfunction
*Ruta graveolens*	Rutaceae	Ruda	Leaves	Childbirth, carminative, menstrual pain, cold in womb
*Scoparia dulcis*	Scrophulariaceae	Sweet broom	Leaves	Prostate
*Urena sinuata*	Malvaceae	Patte chien		"Man's waist pain"
*Vetiveria zizanioides*	Poaceae	Vetivert	Plant	Shorten labour
*Wedelia trilobata*	Asteraceae	Venven caribe	Leaves	Female complaints

**Table 2:** Validation of plants used for reproductive problems in Trinidad and Tobago

Scientific name	Common name	Use	Validation
*Abelmoschus moschatus*	Gumbo musque	Female complaints, remove placenta	2
*Achyranthes indica*	Man better man	Female complaints	3
*Ageratum conyzoides*	Z'herbe á femme	Prostate Womens' complaints	2
*Ambrosia cumanenesis*	Altamis	Inflammation, abortion, Menstrual pain	2
*Aristolochia rugosa*, *Aristolochia trilobata*	Mat root, anico	Remove placenta, abortion, menstrual pain	2
*Artemisia absinthium*	Wormwood	Female complaints	2
*Brownea latifolia*	Cooper hoop	Female complaints	More data needed
*Capraria biflora*	Du thé pays	Menstrual pain	More data needed
*Catharanthus roseus*	White Periwinkle	'Man's disease'	More data
*Chamaesyce hirta*	Malomay	Infertility	3 diuretic
*Clusea rosea*	Matapal	Bark belt for man's waist pain	More data needed
*Cocos nucifera*	Coconut	Abortion	2
*Cola nitida*	Obie seed	Infertility	More data
*Coleus aromaticus*	Spanish thyme	Shorten labour	3
*Commelina elegans*	Water grass	Douche	2
*Cordia curassavica*	Black sage	Menstrual pain	2
*Croton gossypifolius*	Bois sang	Menstrual pain	2
*Cucurbita pepo*, *Cucurbita maxima*	Pumpkin	Prostate problems	2
*Desmodium canum*	Sweet heart bush	Venereal diseases	2
*Eleutherine bulbosa*	Dragon blood	Female complaints	2
*Entada polystachya*	Mayoc chapelle	Menstrual pain	More data
*Eryngium foetidum*	Chadron bénée	Menstrual pain, remove placenta, shorten labour	More data needed
*Eupatorium macrophyllum*	Z'herbe chatte	Womens' complaints	2
*Gomphrena globosa*	Bachelor button	Prostate problems	More data
*Hibiscus rosa-sinensis*	Hibiscus	Female complaints	3
*Justicia pectoralis*	Carpenter grass	Prostate problems	2
*Justicia secunda*	St. John's bush	Womens' complaints	2
*Laportea aestuans*	Red stinging nettle	Shorten labour	More data
*Leonotis nepetaefolia*	Shandileer	Menstrual pain	More data
*Mimosa pudica*	Mese marie	Childbirth	2
*Nopalea cochinellifera*	Rachette	Menopause, hot flashes	2
*Parinari campestris*	Bois bandé	Erectile dysfunction	2
*Parthenium hysterophorus*	White head broom	Womens' complaints	3 pain
*Pilea microphylla*	Du thé bethelmay	Inflammation, womb cleanser	2
*Richeria grandis*	Bois bandé	Erectile dysfunction	2
*Ruta graveolens*	Ruda	Childbirth, carminative, menstrual pain, cold in womb	3
*Scoparia dulcis*	Sweet broom	Prostate	2
*Urena sinuata*	Patte chien	Man's waist pain	2
*Vetiveria zizanioides*	Vetivert	Shorten labour	2
*Wedelia trilobata*	Venven caribe	Womens' complaints	2

### Non-experimental validation of plants used for reproductive problems

*Abelmoschus moschatus *plant is used for reproductive purposes in Fiji [22]. *Abelmoschus manihot *is used for menorrhagia in Vanuatu [23]. Myricetin, a naturally occurring flavonol with antioxidative and cytoprotective proterties is found in *Abelmoschus moschatus *Medic. This flavonol is used in the treatment of depression and anxiety in traditional Chinese medicine and has potential therapeutic benefit for cardiovascular diseases associated with diabetes mellitus [24].

The use of *Achyranthes indica *for venereal diseases in Trinidad has been previously recorded [11]. In Nepal *Achyranthes aspera *is used to facilitate parturition [25]. The benzene extract of the stem bark shows abortifacient activity in the rat [25]. The ethanolic extract of *A. aspera *caused reproductive toxicity in male rats and the action may result from suppressing the synthesis of androgen [26]. The methanolic leaf extract of *Achyranthes aspera *possesses significant (p < 0.05) abortifacient activity and increased pituitary and uterine wet weights in ovarectimized rats. The extract did not significantly influence serum concentration of the ovarian hormones and various lipids except lowering HDL at doses tested [26].

*Ageratum conyzoides *is used for venereal disease in El Salvador [20]. *Ageratum conyzoides *plant extract inhibited uterine contractions induced by 5-hydroxytryptamine suggesting that the extract exhibited specific antiserotonergic activity on isolated uterus plant extract but had no effect on uterine contractions induced by acetylcholine [28,29]. The results support the popular use of the plant as a spasmolytic [28,29].

The use of *Ambrosia cumanenesis *for women's problems has been previously recorded [11]. *Ambrosia elatior *is used as a febrifuge in Tropical America [20]. Dominican healers in New York City use *Ambrosia peruviana *for uterine fibroids [30]. Synonyms of *Ambrosia cumanenesis *are *A. californica*; *A. coronopifolia*; *A. psilostachya *and *Ambrosia rugelii*; these synonyms are provided to guide future researchers to future research; very little is currently available. The sesquiterpene lactone cumanin from *Ambrosia psilostachya*, exerted a high *in vivo *anti-inflammatory response, which was equivalent to that of helenalin (ED_50 _= 6.33 and 13.11 μmol/kg, respectively) [31].

*Aristolochia *species are used in Mexico, western Panama and Guatemala as analgesics, for stomach pain, female disorders, menstrual pain and as contraceptives [11,20]. At doses of 1000 μg/cm2, the chloroform extract of *Aristolochia trilobata*, induced oedema reductions ranging between 50 (bark) and 93% (leaves). The methanol extract produced oedema inhibition of 18% (*A. trilobata *leaves). The chloroform extract of the *Aristolochia trilobata *leaves showed an anti-inflammatory effect comparable to that of indomethacin [32].

*Artemisia absinthium *is used together with other plants as fertility regulators in western Panama and Paraguay and this use is ancient [34,11]. *Artemisia absinthium *is used by the Caribs in Guatemala for fever, vaginitis and stomach pains [11]. Mugwort contains several pain relieving compounds including isothujone, linalool and cineole [35]. These compounds may also relieve premenstrual syndrome. Aqueous extracts of *Artemisia *contain little thujone and are probably safe to use [35].

*Chamaesyce prostrata *was used in Barbados prior to 1834 for venereal complaints [11]. The active component(s) in the water extract of *Euphorbia hirta *leaf have a similar diuretic spectrum to that of acetazolamide [35]. *Chamaesyce hirta *aqueous extract showed central analgesic properties [36].

The antinociceptive action of 13,118-binaringenin (GB-1a), a biflavonoid isolated from *Clusia columnaris *was more potent than some well-known analgesic drugs used as references. Its mechanism of action seems unrelated to the opioid receptors [37]. Compounds isolated from the trunk latex of *Clusia grandiflora *have potent antibacterial activity [38]. A novel antitumoral compound was isolated from *Clusia *spp. resin [39].

*Cocos nucifera *shell produces a fluid when hot that is used ethnomedicinally in India [11]. *Cocos nucifera *is used in complex plant combinations for venereal diseases in Cuba [40]. The fiber husk is rich in catechins with anti-bacterial, anti-viral and anti-proliferative activity on leukaemic cells and normal blood lymphocytes [41].

*In vitro *crude extracts of kola nuts depress smooth muscle activity. The oestrous cycles of rats treated with hydro-alcohol extracts of *Cola nitida*, were blocked at the dioestrous II stage. Only 50% of the cycles of rats treated with *Cola nitida *were disrupted. The extract contains weak antioestrogen-like activity that provokes a blockage of female rat ovulation and oestrous cycle by acting on the hypophysis and/or hypothalamus secretion. This effect was mediated by oestrogen receptors [42].

*Coleus aromaticus *has been used historically for menorrhagia in Trinidad [11].*Coleus barbatus *is used to interrupt pregnancy in Brazil and is used as an emmenagogue in other countries [43].  *Coleus barbatus *showed an anti-implantation effect in the preimplantation period in rats, but after embryo implantation the extract had little effect [43].

*Commelina elliptica *is used in a bath by the Alteños Indians in Bolivia to reduce high fevers. *Commelina pallida *and *C. cayenensis *are used as haemostatics, wound healing and ecbolics in Mexico [11,20]. *Commelina communis *has level 2 validity for diabetes [44]. *Commelina diffusa *extract showed antibacterial and antifungal activity against *Trichophyton *spp., a common dermatophyte [45].

*Cordia alba *was used by the Aztecs as a diuretic. *Cordia spinescens *is used in Colombia to relieve postpartum pain [11].*Cordia boissieri *and *C. collococca *are used as emollient roots in the Antilles and Mexico [20]. *Cordia curassavica *has antibacterial activity against bacteria known to cause gastrointestinal problems [46].

*Croton nepetaefolius *Baill., is used in Brazilian folk medicine as a sedative and antispasmodic agent. *Croton draco*, *C*.*panamensis*, and *C*.*stipulaceus *are used in Mexico for kidney ailments [20]. *Croton berlandieri *bark decoction is used for syphilis in the Yucatan [20]. The essential oil of *C. nepetaefolius*, administered orally, promotes a dose-dependent antinociceptive effect [11,47]. Active principles could include flavonoids and terpenoid compounds [47]. At a dose of 20 mg/kg, intravenous bolus administration of the ethanolic extracts from *Croton schiedeanus *showed significant antihypertensive activity when assayed both in SHR and Wistar rats and in rat isolated aortic rings [48].

*Cucurbita pepo *is used for prostate disorders and urine intermittence in Palestine [11].

*Desmodium gangeticum *is used as an antipyretic in India [11]. The water decoction of root and aerial parts of *Desmodium gangeticum *possesses anti-inflammatory and anti-nociceptive activity. These results support the traditional use of the water decoction of *Desmodium gangeticum *as an analgesic [49].

*Eleutherine bulbosa *is used in Columbia for menstrual cramps and in Haiti as an antifertility agent [10,50]. *Eleutherine *species are used in the Malay Peninsula, Bolivia and Peru for vaginal discharge, wounds, dysentery, diarrhoea and anaemia [11]. *Eleutherine bulbosa *bulb extract showed antifertility and cicatrizant activity and was non-toxic [10].

*Eryngium foetidum *is used in the Caribbean and South America for the treatment of fevers and antiinflammatory disorders and for venereal diseases [11,50]. The topical antiinflammatory activity of the hexane extract and of stigmasterol was established and stigmasterol is not the only bioactive component [51].

*Eupatorium *species are used in South America as contraceptives, abortives and emmenagogues [52]. Two species of *Eupatorium *were used in Trinidad for menstrual problems in 1893 [11]. *Eupatorium macrophyllum *has been used in Trinidad historically for amenorrhoea, dysmenorrhea, prolapse and womb problems in Trinidad [11]. *Chromolaena odorata *(synonym *Eupatorium odoratum*) crude ethanol extract has antioxidant activity that protects fibroblasts and keratinocytes *in vitro *[53]. The phenolic acids present and complex mixtures of lipophilic flavonoid aglycones protected cultured skin cells against oxidative damage [53].

The glycoside isorhamnetin 3-O-beta-robinobioside was found in *Gomphrena boliviana*. Upon inoculation of various doses of 5,6,7-trisubstituted flavones on two murine tumour lines, Sarcoma 180 and Ehrlich's carcinoma, a decrease of tumour growth was observed [54]. *Gomphrena globosa *flowers contain betacyanins which have potential as as food colorants and antioxidants [55].

*Hibiscus rosa-sinensis *flower decoctions are used in India and Vanuatu as aphrodisiacs, for menorrhagia, uterine haemorrhage and for fertility control [11]. Flower extracts produced an irregular estrous cycle in mice with prolonged oestrus and metoestrus and other indications of antiovulatory effects, androgenicity and estrogenic activity [56-60]. *Hibiscus rosa sinensis *possesseses anti-complementary, anti-diarrhetic and anti-phologistic activity. *Hibiscus rosa sinensis *flower showed anti-spermatogenic, androgenic, anti-tumour and anti-convulsant activities [56-60].

*Justicia pectoralis *showed antinociceptive, bronchodilator and antiinflammatory effects [15,11,61]. These activities might be due to the coumarin in the plant [11,15]. One recent review showed that plant species rich in coumarin compounds have potential antineoplastic or cytotoxic activities that are correlated to their use as abortifacients [21].

The leaf and tem decoction of *Leonotis nepetaefolia *is used as an abortifacient and emmenagogue in the Caribbean [52].

*Mimosa pudica *is used in Nicaragua and Mexico for stomach aches, 'cleaning the womb', as a sedative, to stop menstruation and for gonorrhoea [11]. The root is used as a temporary birth control in India [62]. *Mimosa pudica *produced an antidepressant-like profile similar to two tricyclic antidepressants clomipramine and desipramine [63]. Powdered roots were given to adult cycling female albino rats at dosages of 100 mg and 150 mg/kg body weight, administered intragastrically for 5 consecutive days. There was a significant reduction in the number of normal ova in the experimental rats in the study due to inhibition of steroidogenesis, thus producing an imbalance in oestrogen and/or progesterone levels. Alternatively, the powder may have acted on the hypothalamus and reduced the releasing hormones and/or changed the pituitary gonadotropin levels [62].

*Nopalea cochinellifera *is used for pain and inflammation in India [11]. An oral glucose tolerance test showed that stems of *Nopalea cochinellifera *increases blood glucose levels in mice [64].

*Parinari *species are used for venereal diseases in some African countries [11]. The methanolic extract of the stem bark of *Parinari polyandra *demonstrated anti-nociceptive and anti-inflammatory effects in mice and rats, justifying the local use of the plant [65].

*Parthenium hysterophorus *is used as a tonic, analgesic, antipyretic, antiperiodic, febrifuge and emmenagogue in Mauritius, Rodrigues, Mexico, Belize and India [11,20]. It is used for venereal diseases in Cuba [40]. A depolarizing neuromuscular junctional blocking action of *Parthenium hysterophorus *leaf extracts was found in the rat [66].

*Pilea microphylla *is used ethnomedicinally in Asia and in Central America [11,20]. The entire plant is given to women in labour in Jamaica. *Pilea microphylla *was active against *Staphyloccocus aureus *[67].

*Richeria grandis *(syn. *Guarania ramiflora*) is used as an aphrodisiac in Trinidad. *Roupala montana *is also used in Trinidad and is a documented nervine [68].

*Ruta graveolens *and closely related species are used as emmenagogues, abortives, antispasmodics, sudorifics and anthelmintics in France, Spain, Brazil, Paraguay, New Mexico, Italy, Madeira and in other cultures and the antifertility uses were documented by Galen and Pliny the Elder [69,70,11]. *Ruta *species contain different alkaloids and furanocoumarins and may show toxic side effects when used as abortifacients [11,69]. *Ruta graveolens *has shown weak activity *in vitro *on excised uterine muscle [70]. The antimicrobial activity of the plant is possibly due to the essential oils or flavonoids [71]. Data collected in Uruguay from 1986 – 1999 of 86 cases of abortion involved 30 different plant species [72]. *Ruta chalepensis *or *Ruta graveolens *were among the species most frequently used for abortions [72]. Multiple organ system failure occurred in some patients who had ingested ruda (alone or in combination with parsley or fennel). Deaths occurred in 4 cases of ruda ingestion (2 cases of ruda alone, 2 cases of ruda with parsley and fennel) [72].

*Scoparia dulcis *is used in Nicaragua for belly pain and to 'clean the blood, kidney and system' [11]. Antitumour-promoting compounds and antiviral agents were found in *Scoparia dulcis *[22]. It also has antimicrobial and antifungal effects as well as antihyperlipidemic action in normal and experimental diabetic rats in addition to its antidiabetic activity [73].

*Urena sinuata *plant is used for reproductive purposes in the Pacific, Trinidad, China and India [11,22]. Its synonyms are *Urena aculeata *Mill., *U. lobata *ssp. *sinuata *(L.) Borss. Waalk., *Urena morifolia *DC., *U. muricata *DC., *U. paradoxa *Kunth and *U. swartzi*. The methanol extract of *Urena lobata *root exhibited broad spectrum antibacterial activity [74].

*Vetiveria zizanioides *is used in Pakistan as an emmenagogue and stimulant and is used by the Caribs in Guatemala for stomach pains [11].

*Wedelia trilobata *has been historically used for amenorrhea in Trinidad [11]. Kaurenoic acid and luteolin in *Wedelia paludosa *showed antinociceptive action more potent than the standard analgesic drugs (acetyl salicylic acid, acetaminophen, dipyrone and indomethacin) [75]. *Wedelia paludosa *and *Wedelia trilobata *contain the diterpene (kaurenoic acid), eudesmanolide lactones and luteolin (in leaves and stems) [11,75]. Kaurenoic acid has antibacterial, larvicidal and tripanocidal activity; it is also a potent stimulator of uterine contractions [75]. Luteolin exerts antitumoural, mutagenic and antioxidant effects, has depressant action on smooth muscles and a stimulant action on isolated guinea pig heart [75].

## Discussion and Conclusion

In 1859 Victorian novelist Anthony Trollope made the following observation:

As Trinidad is an English colony, one's first idea is that the people speak English; and one's second idea, when that other one as to the English has fallen to the ground, is that they should speak Spanish, seeing that the name of the place is Spanish. But the fact is that they all speak French [11].

The plants discussed in this paper reflect the historical fact that Trinidad was first a Spanish then a British colony. Antonio de Sedeño first settled Trinidad in the 1530s as a means of controlling the Orinoco and subduing the Warao. Spanish colonisation in Trinidad remained tenuous. In 1762, after three hundred years of Spanish rule San José de Oruña and Puerto España (Port of Spain) were hamlets rather than towns. Because Trinidad was considered underpopulated, Roume de St. Laurent, a Frenchman living in Grenada, was able to obtain a Cédula de Población from the Spanish King Charles III on the 4th November, 1783 [11]. This Cédula de Población was more generous than the first of 1776 and granted free lands to Roman Catholic foreign settlers and their slaves in Trinidad willing to swear allegiance to the Spanish king. The land grant was thirty two acres for each man, woman and child and half of that for each slave brought. As a result, Scots, Irish, German, Italian and English families arrived. The French Revolution (1789) also had an impact on Trinidad's culture since it resulted in the emigration of Martiniquan planters and their slaves to Trinidad who established an agriculture-based economy (sugar and cocoa) for the island.

The population of Puerto de España (Port of Spain) increased from under 3,000 to 10,422 in five years and the inhabitants in 1797 consisted of mixed-races, Spaniards, Africans, French republican soldiers, retired pirates and French nobility. The small towns of Siparia and Arima were established by the Spanish Capuchins who came from the Santa Maria province of Aragon in 1756 – 1758.

The name *semen contra *now used in the West Indies as the Creole name for the introduced European plant *Chenopodium ambrosioides *was originally one of the names of the drug Santonica derived from the introduced European plant *Artemesia cina *B. *Artemesia *was also used as an anthelmintic but perhaps less effectively. Spanish traditions have probably been handed down from the original colonial heritage but are reinforced by visits and migrants escaping the turbulent politics of Venezuela.

*Artemisia absinthium *is used together with other plants as fertility regulators by the French, Spanish New Mexicans (emmenagogue) and in Madeira and this use is ancient [11]. In the 1800s *Ageratum conyzoides *was called 'herbe chatte' and *Eupatorium ayapana *was 'z'herbe à femme'. A name change in the last century may have occurred because of the use of *Ageratum conyzoides *then and currently (given to women after childbirth and to promote menstruation) [11].

Hispanic prayers are used in Latin America for healing and against mal yeux. These spanish-romanic prayers, like the 'oracion' prayer are used during 'santowah' (santigual. The ceremony includes sweet broom (*Scoparia dulcis*) which is used to sprinkle holy water (S. Moodie-Kublalsingh, Institute of Languages, University of The West Indies, pers. comm. August, 2000). These prayers (magic rather than religion) are said to have come to the New World with the conquistadors.

The non-experimental validation method can be used to advise the public on which plants are safe, effective and useful, and which are not; pending clinical trials. Studies conducted on *Ruta graveolens *indicate that there are safety issues that need addressing.

The plants used for reproductive problems have some support. *Chamaesyce hirta *has scientific support but as a diuretic. Other plants with level 3 validity are: *Achyranthes indica*, *Coleus aromaticus*, *Hibiscus rosa-sinesis*, *Parthenium hysterophorus *and *Ruta graveolens*. Plants that have limited support are: *Abelmoschus moschatus*, *Ageratum conyzoides. Ambrosia cumanenesis*, *Aristolochia rugosa*, *Aristolochia trilobata*, *Artemisia absinthium*, *Cocos nucifera*, *Commelina elegans*, *Cordia curassavica*, *Croton gossypifolius*, *Cucurbita pepo*, *Cucurbita maxima*, *Desmodium canum*, *Eleutherine bulbosa*, *Eupatorium macrophyllum*, *Justicia pectoralis*, *Justicia secunda*, *Mimosa pudica*, *Nopalea cochinellifera*, *Parinari campestris*, *Pilea microphylla*, *Richeria grandis*, *Scoparia dulcis*, *Urena sinuata*, *Vetiveria zizanioides*, and *Wedelia trilobata*. Plants that have little research data are: *Brownea latifolia*, *Capraria biflora*, *Catharanthus roseus*, *Clusea rosea*, *Cola nitida*, *Entada polystachya*, *Eryngium foetidum*, *Gomphrena globosa*,*Laportea aestuans*, and *Leonotis nepetaefolia*.

In a previous study conducted in 1995 the red flowers of forest tree called cooper hook (*Brownea latifolia*) were given for women's menstrual problems, gripes and pain. A decoction of the flowers is red in colour [76]. A male informant whose mother was a midwife used both *Brownea latifolia *and red monkey step vine (*Bauhinia cumanensis */*Bauhinia excisa*) to make tisanes for women's problems. Apparently using the Doctrine of Signatures, he insisted that the vine when cut should not be white in colour but red like blood. This tisane he claimed would clean out women's insides preventing monthly period pain and would also improve fertility.

The main categories of the Doctrine of Signatures are: similarity between the substance used and the human organ; resemblance in shape or behaviour to a specific animal; correlation between the colour of a substance and the colour of the symptoms; similarities between the substance and the patient's symptoms and the use of a substance that might produce symptoms of a particular disease in a healthy person to remedy those same symptoms in one who is sick [77]. The use of this Doctrine in Trinidad may have many origins including Amerindian. The Waorani in Amazonian Ecuador have a similar logic [78].

The Caribbean's first recognised medicinal plant for reproductive and other problems was lignum vitae (*Guaiacum officinale*) [11]. In 1603, Bartholomew Gilbert set sail for Nevis from London in the 50-ton Elizabeth, to cut lignum vitae and buy tobacco from the Amerindians [11]. This long-known Caribbean plant has had very little scientific study however, similarly to the other native Caribbean plants evaluated in this paper. This limits the utility of the non-experimental validation of the plants; however the validation provides available information until clinical trials are done.

Caribbean folk medicine has been influenced by European folk medicine, either through the early Spanish and French settlers or through the continuous immigration of Spanish-speaking peoples from Venezuela. Ruda (*Ruta graveolens*) is a plant of European origin and closely related species are used as emmenagogues, abortives, antispasmodics, sudorifics and anthelmintics across Europe. The antifertility uses were documented by Galen and Pliny the Elder. *Artemisia absinthium *is also used across Europe for reproductive purposes and these uses are ancient [11,79].

There is currently a commission of inquiry into the operations and delivery of public healthcare services in Trinidad and Tobago [80]. Health care cooperatives in Costa Rica provide an example of how medical costs could be controlled in Trinidad if a similar program was followed [81]. In the 1990s, the cooperatives conducted 9.7–33.8% more general visits, 27.9–56.6% more dental visits, and 28.9–100% fewer specialist visits. Real total expenditure per capita in cooperatives was 14.7–58.9% lower than in traditional clinics [81]. Trinidad has also required and benefited from the skills of visiting Cuban doctors and other foreign doctors. International solidarity has always been at the centre of the Cuban societal project and it has come to the aid of other Caribbean basin countries including Haiti and Venezuela [82]. Rather than importing generalists, if there were more female trained paraprofessionals in place; specialists could be brought in to serve short-term needs. The Jamaican model of expanding the role of community midwives could be followed in Trinidad. Additionally community-based co-ops that include non-traditional practioners, midwives and validated folk-medicine could be studied as an innovative answer to Trinidad and Tobago's on-going health problems.

## Competing interests

The author(s) declare that they have no competing interests.
